# Adjunctive treatment with moxonidine versus nitrendipine for hypertensive patients with advanced renal failure: a cost-effectiveness analysis

**DOI:** 10.1186/1471-2369-8-9

**Published:** 2007-07-24

**Authors:** Kavi J Littlewood, Wolfgang Greiner, Dominique Baum, York Zoellner

**Affiliations:** 1 Mapi Values Netherlands BV, Houten, The Netherlands; 2 Universität Bielefeld, Fakultät für Gesundheitswissenschaften, Bielefeld, Germany; 3 Solvay Pharmaceuticals GmbH, Hannover, Germany

## Abstract

**Background:**

Systemic hypertension often accompanies chronic renal failure and can accelerate its progression to end-stage renal disease (ESRD). Adjunctive moxonidine appeared to have benefits versus adjunctive nitrendipine, in a randomised double-blind six-month trial in hypertensive patients with advanced renal failure. To understand the longer term effects and costs of moxonidine, a decision analytic model was developed and a cost-effectiveness analysis performed.

**Methods:**

A Markov model was used to extrapolate results from the trial over three years. All patients started in a non-ESRD state. After each cycle, patients with a glomerular filtration rate below 15 ml/min had progressed to an ESRD state.

The cost-effectiveness analysis was based on the Dutch healthcare perspective. The main outcome measure was incremental cost per life-year gained. The percentage of patients progressing to ESRD and cumulative costs were also compared after three years. In the base case analysis, all patients with ESRD received dialysis.

**Results:**

The model predicted that after three years, 38.9% (95%CI 31.8–45.8) of patients treated with nitrendipine progressed to ESRD compared to 7.5% (95%CI 3.5–12.7) of patients treated with moxonidine. Treatment with standard antihypertensive therapy and adjunctive moxonidine was predicted to reduce the number of ESRD cases by 81% over three years compared to adjunctive nitrendipine.

The cumulative costs per patient were significantly lower in the moxonidine group €9,858 (95% CI 5,501–16,174) than in the nitrendipine group €37,472 (95% CI 27,957–49,478).

The model showed moxonidine to be dominant compared to nitrendipine, increasing life-years lived by 0.044 (95%CI 0.020–0.070) years and at a cost-saving of €27,615 (95%CI 16,894–39,583) per patient.

Probabilistic analyses confirmed that the moxonidine strategy was dominant over nitrendipine in over 98.9% of cases. The cumulative 3-year costs and LYL continued to favour the moxonidine strategy in all sensitivity analyses performed.

**Conclusion:**

Treatment with standard antihypertensive therapy and adjunctive moxonidine in hypertensive patients with advanced renal failure was predicted to reduce the number of new ESRD cases over three years compared to adjunctive nitrendipine. The model showed that adjunctive moxonidine could increase life-years lived and provide long term cost savings.

## Background

End-stage renal disease (ESRD) is a disorder that occurs worldwide and is associated with a high cost to society due to the need for dialysis or renal transplantation. In 1994, 7,340 patients in the Netherlands were receiving renal replacement therapy, incurring direct medical costs of NLG 584 million (262 million 1994 €) and indirect costs of NLG 3.5 million (1.6 million 1994 €). De Wit et al estimated that in 2003, there would be 11,500 patients receiving renal replacement therapy at a cost to society of over NLG 900 million (405 million 1994 €) [1].

Systemic hypertension frequently accompanies chronic renal failure (CRF) and is a strong risk factor for the development of ESRD [2]. Thus current drugs target systemic hypertension in addition to proteinuria in an attempt to slow the progression of renal disease [3]. Angiotensin-converting enzyme inhibitors (ACEI) or angiotensin receptor blockers (ARB) are the treatment of choice for hypertensive patients with CRF, with the addition of diuretics for inadequate blood pressure control. Patients often require athird drug to control hypertension effectively [4,5].

Moxonidine may be a good candidate for adjunctive treatment to standard therapy as it interferes with the sympathetic nervous system overactivity [6] which may contribute to hypertension in CRF patients [6-9].

The short term benefits of adjunctive treatment with moxonidine on top of standard antihypertensive therapy have been shown, versus adjunctive nitrendipine, in a single randomised double-blind 24-week trial in hypertensive patients with advanced renal failure. Most patients received standard therapy of ACEI or ARB plus diuretics. Although this study was primarily a safety and tolerability study, a significant difference was seen in creatinine clearance decline between the moxonidine group and the comparator group after 24 weeks of treatment [10]. This was the only study found comparing moxonidine to nitrendipine in a review of the literature to date. Creatinine clearance is an indicator of the glomerular filtration rate of the kidneys. A reduction in creatinine clearance levels indicates a decline in kidney function. The trial measured baseline proteinuria in around 60% of patients in each group and found levels were similar across both groups. But proteinuria levels were not available in 40% of patients and therefore it is not possible to know this impact on the overall trial outcomes, as proteinuria is a risk factor for renal disease progression. Therefore a major assumption of our study was that the differences in creatinine clearance decline were not caused by other risk factors (such as underlying disease or proteinuria level). Based on this assumption, it was hypothesised that adjunctive moxonidine in hypertensive patients with renal failure may contribute to decreasing the burden of ESRD.

In order to understand the effects of moxonidine on renal progression and on costs over a longer period, a decision analytic model was developed and a cost-effectiveness analysis was performed on the basis of the Dutch healthcare setting.

## Methods

### Decision-Analytic Model and assumptions

**Figure 1:**
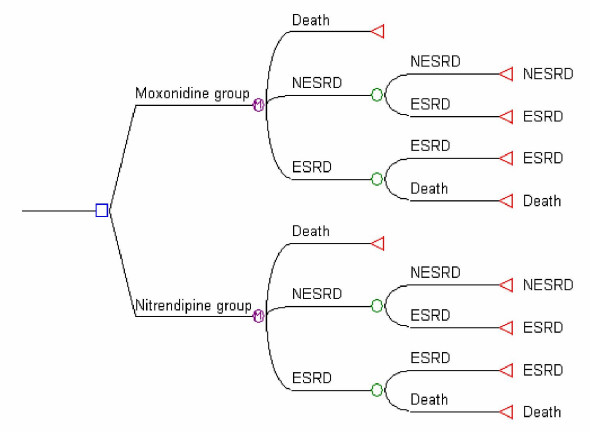
The Decision analytic model. Legend □ Decision node  Markov node ○ Choice node ◁ Terminal node

A Markov model was built in MS Excel to extrapolate results from the six-month trial, of adjunctive moxonidine versus adjunctive nitrendipine, over three years (or six cycles of six months). This time horizon of three years closely approximates the maximum follow-up period of cohorts of ESRD patients found in the literature [11-14]. In the model, all patients started in a non-ESRD state (NESRD). NESRD was defined as patients with advanced renal failure and hypertension, with a glomerular filtration rate (GFR) above 15 ml/min, not treated with dialysis or transplant. After each cycle of six months, patients with a GFR below 15 ml/min were considered to have progressed to the ESRD state. This cut-off value of 15 ml/min was based on the European Renal Association (ERA) guidelines [15] and US National Kidney Foundation guidelines [16]. Once in the ESRD state, patients could not go back to the NESRD state, they could either remain in this state or progress to death. (Figure 1)

Creatinine clearance measures from the trial were not measured directly, but were obtained using the Cockcroft & Gault equation, and assumed to equal GFR measures in the model. The Cockcroft-Gault equation is frequently used for estimating GFR in adults. Although it was originally developed for estimating creatinine clearance, it has been widely tested as a good predictor of GFR [17]. Age, gender, and body weight are used to correct for the differences in muscle mass, and hence creatinine generation rate.

### Transition probabilities

Transition probabilities for moving from the NESRD (GFR > 15 ml/min) to ESRD (GFR < 15 ml/min) state were based on extrapolations of the trial results. The average GFR decline over the three year time horizon was assumed to be linear. A linear progression was judged to be acceptable in these very renally-impaired patients. The mean decline in GFR over the trial period – from baseline (visit 2) to week 24 (visit 9) – was 0.7 ml/min for the moxonidine group and 1.8 ml/min for the nitrendipine group. This average decline over a six-month period was applied to each six-month period in the model, with accompanying standard deviations (SD) keeping the ratio of mean/SD constant throughout the cycles. Based on the given and estimated means and SD, the percentage of patients with ESRD (GFR < 15 ml/min) was then calculated for each six-month period using the gamma distribution (parameters Moxonidine α = 1.445, β = 83.555, Nitrendipine: α = 12.403, β = 66.597). The gamma distribution was found to best describe the trial data compared to the normal or lognormal distribution (skewed to the right with no negative values) [18,19]. A consequence of this method is that there were some patients with ESRD at baseline in both groups, and these patients were not receiving dialysis or transplants. Therefore only new cases of ESRD were used to derive transition probabilities for the model.

For each six-month period: *Transition probability = new cases ESRD/NESRD population *(Table 1.)

**Table 1:** Transition probabilities of moving from NESRD state to ESRD state

Cycle	NESRD to ESRD^†^		NESRD to Death^††^	ESRD to Death^‡^			
	moxonidine	nitrendipine	all-cause	Dialysis	Transplant	Dial/Trans 64%/34%	Dial/Trans 50%/50%

1	0.019	0.077	0.0042	0.067	0.013	0.048	0.040
2	0.012	0.053	0.0042	0.067	0.013	0.048	0.040
3	0.013	0.069	0.0042	0.067	0.013	0.048	0.040
4	0.014	0.089	0.0042	0.067	0.013	0.048	0.040
5	0.016	0.118	0.0042	0.067	0.013	0.048	0.040
6	0.017	0.157	0.0042	0.067	0.013	0.048	0.040

^†^Based on data from Vonend *et al *2003.

^††^Based on Dutch all-cause mortality 2005 (8.4 per 1,000 inhabitants), (Centraal Bureau voor de Statistiek)

^‡^Based on data from ERA EDTA Annual report 2002.

Transition probabilities for moving from the ESRD state to Death were based on Dutch mortality rates for patients on dialysis and transplanted patients. The base case model assumed all patients were on dialysis. As survival rates were very different for ESRD patients on dialysis or receiving transplants, the percentage of patients on each of these types of renal replacement therapy had to be varied in sensitivity analyses[20]. (Table 1.)

### Summary of assumptions

• A time horizon of 3 years approximates the maximum follow-up period of ESRD patients

• The difference in GFR decline found in the trial was not caused by differences in other risk factors for decline in renal function (like underlying disease or proteinuria level).

• The Cockroft and Gault formula is acceptable to calculate the GFR levels for the population of the study.

• The average decline in GFR over the years can be described by a linear function

• Extrapolating the average decline found over the initial 6 months will not overestimate the decline in the following periods.

• The gamma distribution is best to estimate the percentage of patients with a GFR < 15.

• All patients received dialysis during the ESRD state.

• From the NESRD state patients could stay in this state or enter the ESRD state

• From the ERSD state patients could stay in this state or enter the death state but they could not go back to the NESRD state.

### Resource use and costs by health state

Costs per cycle for the NESRD state included general medical costs (consultations, diagnostics and laboratory services) based on costing data from the economic evaluation of benazepril for Dutch CRF patients [21], and drug usage from the trial (moxonidine, nitrendipine and an average cost of hypertension drugs used) [10]. Diuretic drug costs were assumed to be covered in the general medical costs. The ESRD state costs per cycle included costs for dialysis or transplantation, consultations, general drug, diagnostic and laboratory services costs based on the economic evaluation of ESRD treatments in the Netherlands performed by the Wit et al[22]. Costs associated with entering the state Death were based on costs for patients in the terminal phase of ESRD reported by van Hout et al [21]. (Table 2.)

**Table 2:** Breakdown of costs (€) by health state.

Cost components per cycle (6-months)	NESRD	ESRD First 2 cycles	ESRD Subsequent cycles	Terminal ESRD
moxonidine 0.3 mg/d^†^	82.80	-	-	-
nitrendipine 20 mg/d^†^	74.40	-	-	-
Other antihypertensives^†^	93.90	-	-	-
Consultations, diagnostics, laboratory services, diuretics^‡^	213.00	-	-	-
Dialysis including consultations, drugs, diagnostics and laboratory services*	-	34,522.00	32,627.00	-
Transplantation including consultations, drugs, diagnostics and laboratory services*	-	22,850.00	4,570.00	-
Terminal ESRD care^‡^	-	-	-	1,416.00

† z-index: Cost of antihypertensives based on patient usage: 89% on ACE I (ddd enalapril 25 mg) and 11% on ARB (ddd losartan 50 mg), and 35.6% on beta blocker (ddd atenolol 75 mg) and 27.4% on alpha blocker (ddd doxazosin 4 mg). Costs of diuretics were assumed to be included in NESRD costs based on manuscript van Hout et al.

‡ van Hout et al

* de Wit et al

All cost data were expressed in Euros; USD costs were converted to Euro by purchasing power parities [23]. All costs were transformed to 2004 prices by means of price indexes from the Dutch "Manual for cost calculations: methods and recommend prices for economic evaluations in health care." All drug costs were based on public prices (ex VAT) from the Dutch Z-index. The daily defined dosage was used to calculate the drug price per cycle.

Generally Markov models assume transitions from one health state to the next occur at the end of the cycle, however in reality this transition can occur at any point during the cycle, therefore a half-cycle correction was incorporated.

### The cost-effectiveness analysis

The cost-effectiveness analysis was based on the Dutch healthcare perspective, with a time horizon of three years from the start of treatment. The main outcome measure was the incremental cost per life-year gained. The percentage of patients with ESRD and the cumulative costs were also compared at three years. A discount rate of 4% on costs and 1.5% on life-years per annum was applied, as recommended by the Dutch guidelines [24].

The cost-effectiveness analysis was performed for a hypothetical cohort of patients similar to the patients in the trial: adult patients with advanced renal failure (GFR < 30 ml/min) and hypertension (DBP 80–100). Patients were already receiving treatment with loop diuretics and ACEI or ARB, and were given moxonidine (0.3 mg/day) or nitrendipine (20 mg/day). Patient exclusion criteria included presence of sick sinus syndrome, higher degree sino-atrial or atrioventricular block, bradycardia, malignant arrhythmia, heart failure of New York Heart Association stage III or IV, severe chronic ischemic heart disease, symptomatic cerebrovascular disease, unstable angina pectoris, severe hepatic disease, history of malignant disease within two years, pregnancy or lactation, history of depression, drug abuse or alcoholism.

### Scenario Analyses

In the base case analysis, all patients with ESRD were assumed to receive dialysis treatment, a discount rate was applied to costs and benefits, a linear decline was assumed for progression to ESRD and no patients died from the NESRD state. Scenario analyses were performed to evaluate the effects and costs if:

• 64% of patients received dialysis treatment and 36% received a transplant (split based on the incidence figures of the Dutch Renine Registry for 2002 [20])

• or 50% of patients received dialysis and 50% received a transplant (split based on prevalence figures from the Dutch Renine Registry 2002 [20])

Both analyses were performed with a 'living donor to cadaver donor' ratio of 34% to 66% [20].

• 0% discount on costs and benefits was applied

• an exponential decline was used for progression to ESRD

• all-cause Dutch death rate was applied to patients with NESRD

### Probabilistic sensitivity analyses

The source data are characterised by uncertainty. A probabilistic sensitivity analysis was performed to quantify the uncertainty in the model outcomes. A random value was repeatedly sampled from distributions reflecting the uncertainty level of the input source data, plugged into the model, and then the outcome of the model was calculated. Each model outcome is presented with a point estimate along with uncertainty reflected by the 2.5 th and 97.5th percentile of the uncertainty distribution. Uncertainty in the transition probabilities from NESRD to ESRD were based on the trial and expressed with beta distributions (alpha and beta determined from the standard error). Uncertainty in resource data were expressed with triangular distributions, with the low and high values as 80% and 120% of the expected value. Probabilistic sensitivity analysis was performed for both the base case scenario and for scenario analyses.

## Results

### Effects

**Figure 2:**
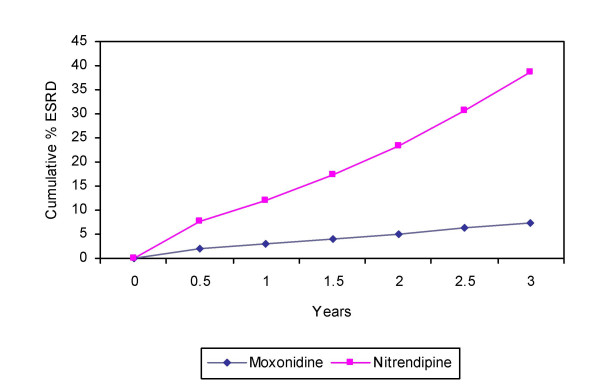
Cumulative percent of patients with ESRD after 3 years.

The model predicted that after three years, 38.9% (95%CI 31.8–45.8) of patients treated with nitrendipine progressed to ESRD compared to only 7.5% (95%CI 3.5–12.7) of patients treated with moxonidine. Thus suggesting that standard treatment with adjunctive moxonidine could prevent 31.4% of new cases of ESRD compared to adjunctive nitrendipine (see Figure 2.), or reduce the number of ESRD cases by 81%.

The proportion of survivors was 98.6% in the moxonidine group after three years compared to 93.9% in the nitrendipine group. Thus the model suggests that patients treated with moxonidine could gain on average 0.044 (95%CI 0.020–0.070) life-years compared to patients on nitrendipine (discounted life-years were 2.950 (95%CI 2.937–2.959) versus 2.907 (95%CI 2.882–2.927).

### Costs

**Figure 3:**
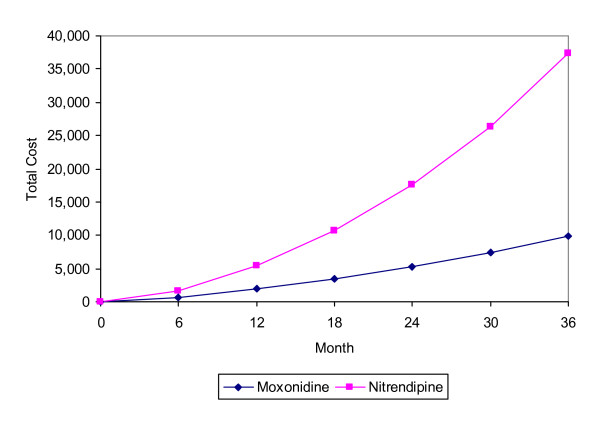
Expected 3-year cumulative cost per patient.

After three years, the cumulative costs per patient appeared to be lower in the moxonidine group (€9,858 with 95% CI 5,501–16,174) than in the nitrendipine group (€37,472 with 95% CI 27,957–49,478) (see Figure 3). Thus moxonidine provided a cost saving of €27,615 (95%CI 16,894–39,583).

### Cost-effectiveness

The model calculated that moxonidine treatment provided a cost saving of €27,615 (95%CI 16,894–39,583) and extra life-years gained of 0.044 (with 95% CI 0.020–0.070) years compared to nitrendipine treatment, thus moxonidine was the dominant strategy. (Table 3) Moxonidine is said to be dominant because it could provide a more effective treatment strategy and for less cost. Probabilistic sensitivity analyses confirmed that the moxonidine strategy was dominant over nitrendipine in over 98.9% of cases.

**Table 3:** Results of the base case model after 3 years

	% progress to ESRD (95%CI)	Life years lived (95%CI)	Costs (€) (95%CI)
Moxonidine	7.5% (3.5–12.7)	2.950 (2.937–2.959)	€9,858 (5,501–16,174)
Nitrendipine	38.9% (31.8–45.8)	2.907 (2.882–2.927)	€37,472 (27,957–49,478)

### Costs and impact of treatment

Thus if 100 patients were given each treatment over three years, the model predicts that after three years 7 patients on moxonidine versus 39 on nitrendipine will have progressed to ESRD, and 1 patient will have died in the moxonidine arm versus 6 in the nitrendipine arm. The cost of treating the moxonidine group would be €985,800 versus €3,747,200 for the nitrendipine group, due to the high cost of treating patients who have progressed to ESRD.

### Scenario analyses

The proportion of ESRD patients receiving dialysis or transplantations was varied in sensitivity analyses. The model showed that when 64% of patients received dialysis (and 34% received transplantations), the cumulative 3-year costs remained lower with moxonidine versus nitrendipine (€8,496 (95% CI 4,920–13,367) versus €31,035 (95% CI 23,618–40,163) respectively), and life-years remained higher in the moxonidine arm (2.954 (95% CI 2.944–2.960) versus 2.923 (95% CI 2.907–2.936) respectively). When 50% of patients received dialysis (and 50% transplantation), the cumulative 3-year costs and LYL continued to favour the moxonidine strategy versus nitrendipine (€7,953 (95% CI 4,603–12,546) and 2.955 (95%CI 2.946–2.960) for moxonidine versus €28,649 (95% CI 21,938–36,392) and 2.929 (95% CI 2.915–2.941) for nitrendipine).

When a 0% discount rate was applied to costs and effects the following results were found: Cumulative costs were €10,349 (95%CI 5,572–17,282) with moxonidine and €39,035 (95% CI 29,324–50,003) with nitrendipine, resulting in a cost saving of €28,686 (95% CI 17,772–41,347) with moxonidine. Life-years lived were 2.987 (95% CI 2.971–2.996) for moxonidine and 2.943 (95% CI 2.919–2.963) for nitrendipine, resulting in 0.044 (95% CI 0.019–0.070) life-years gained with moxonidine.

When the average GFR decline was assumed to decrease exponentially, the results still supported moxonidine over nitrendipine; all patients on nitrendipine had progressed to ESRD after 2 years versus 25% of patients on moxonidine (or 74% after 3 years). Thus moxonidine would provide 0.084 (95%CI 0.059–0.108) additional life-years at a cost saving of €48,364 (95%CI 37,966–59,198) after 3 years.

When Dutch all-cause mortality was incorporated to the NESRD arm, the life-years gained with moxonidine was 0.041 (95%CI -0.035–0.112) years at a cost saving of €27,252 (95%CI 16,652–38,854). Moxonidine was dominant in 90% of cases.

## Discussion

With this model, moxonidine treatment on top of standard therapy in severely renally-impaired patients appears to be cost-effective versus adjunctive nitrendipine, resulting in lower treatment costs (€27,615 lower (95%CI 16,894–39,583)) and a greater number of life-years lived 0.044 (with 95% CI 0.020–0.070) when the results of the six month trial are extrapolated over three years. Sensitivity analyses have shown that these results are robust. Thus given the assumptions of the model, we can conclude that moxonidine could delay progression to ESRD in a large number of patients, thereby reducing the numbers of patients requiring dialysis or transplants compared to standard treatment with adjunctive nitrendipine. Treatment with moxonidine could therefore contribute to reducing the economic burden of ESRD and allow patients to live longer in a non-ESRD state.

### Limitations of the model

Data used in the model was taken from the only study available at the time of moxonidine versus nitrendipine, which was a six-month trial, in 171 patients, with proteinuria levels measured in 103 patients. In the trial, creatinine clearance was calculated using the Cockcroft & Gault equation. As creatinine clearance is the result of clearance due to GFR and secretion of creatinine in the tubules, the model assumed that both treatments had no effect on secretion, thus GFR was equated to creatinine clearance measures alone.

The difference in GFR decline in the trial was assumed not to be due to differences in risk factors for renal progression such as level of proteinuria like albuminuria. Data on albuminuria were only available for a subset of patients in the trial (52/89 moxonidine patients and 51/82 nitrendipine patients). These data showed no significant differences between groups, although mean and median albuminuria values were lower in the moxonidine group (1.3 ± 1.8; 0.8 versus 1.9 ± 2.1; 1.1 at baseline). A limitation of this study is that the missing data for the remaining patients may mean that the two groups were not well matched for albuminuria, a major prognostic factor for renal decline [10].

The average GFR decline over the three year time horizon was assumed to be linear. This is not uncommon in the literature [25-27]. The extrapolation of GFR values from the six month trial also implied that the decline in the first three months after starting treatment was not significantly different than the decline in the following periods, as is sometimes suggested in the literature [18]. However some clinicians assume an exponential decline over time. If the decline is exponential, and disease progression occurs faster, the benefit of moxonidine will be even greater as it appears to delay progression compared to nitrendipine. The results of the scenario analysis show that all nitrendipine patients would have progressed to ESRD compared to 25% of moxonidine patients after two years.

The model only included direct costs. The addition of indirect costs would however be expected to increase the benefit seen with moxonidine, as patients remain in a better state of health for longer and are therefore able to be more productive.

Patients with ESRD are presumed to have much worse quality of life than patients at earlier stages of the disease. Utility values found in the literature report measures of 0.41 for patients on kidney dialysis [28] compared to 0.60 for patients with chronic renal failure [29]. A higher utility value is associated with a better state of health. As such, a cost-utility analysis would be expected to produce even better results for moxonidine compared to standard treatment with nitrendipine, as patients treated with moxonidine remained in a non-ESRD state for longer.

Adjunctive treatment with moxonidine was assumed to have a renoprotective effect occurring independently of blood pressure control in the six month trial. This effect may be supported by the results of a recent study in which moxonidine was the only factor that significantly reduced the risk of a decrease of 50% or more in creatinine clearance rate, or the need for dialysis, or death, in patients following renal transplant. The relative risk reduction due to moxonidine was 0.3 (95% CI 0.1 – 0.6) in the multivariate analysis [30]. Results of a small study in the Netherlands may also support the benefits of adjunctive moxonidine in chronic renal failure. In this study, moxonidine normalised sympathetic hyperactivity, which plays a role in renal hypertension, in patients on chronic eprosartan treatment [19].

## Conclusion

The model extrapolated results from a 6-month trial over three years. Treatment with standard antihypertensive therapy (ACE inhibitor or ARB and loop diuretics) and adjunctive moxonidine in hypertensive patients with advanced renal failure was predicted to reduce the number of ESRD cases by 81% over three years compared to adjunctive nitrendipine. The model showed moxonidine to be dominant compared to nitrendipine, increasing life-years lived by 0.044 (95%CI 0.020–0.070) years and at a cost-saving of €27,615 (95%CI 16,894–39,583) per patient.

## Competing interests

This study was sponsored by Solvay Pharmaceuticals GmbH, Hannover, Germany.

## Authors' contributions

KL, YZ and DB contributed to the design of the study, data collection and analysis. KL and WG contributed to the drafting and reviewing of the manuscript. All authors read and approved the final manuscript.

## Pre-publication history

The pre-publication history for this paper can be accessed here:


